# α7 Nicotinic Acetylcholine Receptor-Specific Antibody Induces Inflammation and Amyloid β_42_ Accumulation in the Mouse Brain to Impair Memory

**DOI:** 10.1371/journal.pone.0122706

**Published:** 2015-03-27

**Authors:** Olena Lykhmus, Larysa Voytenko, Lyudmyla Koval, Sergiy Mykhalskiy, Victor Kholin, Kateryna Peschana, Marios Zouridakis, Socrates Tzartos, Sergiy Komisarenko, Maryna Skok

**Affiliations:** 1 Palladin Institute of Biochemistry, Kyiv, Ukraine; 2 Bogomoletz Institute of Physiology, Kyiv, Ukraine; 3 Chebotaryov Institute of Gerontology, Kyiv, Ukraine; 4 Hellenic Pasteur Institute, Athens, Greece; University of Leipzig, GERMANY

## Abstract

Nicotinic acetylcholine receptors (nAChRs) expressed in the brain are involved in regulating cognitive functions, as well as inflammatory reactions. Their density is decreased upon Alzheimer disease accompanied by accumulation of β-amyloid (Aβ_42_), memory deficit and neuroinflammation. Previously we found that α7 nAChR-specific antibody induced pro-inflammatory interleukin-6 production in U373 glioblastoma cells and that such antibodies were present in the blood of humans. We raised a hypothesis that α7 nAChR-specific antibody can cause neuroinflammation when penetrating the brain. To test this, C57Bl/6 mice were either immunized with extracellular domain of α7 nAChR subunit α7(1-208) or injected with bacterial lipopolysaccharide (LPS) for 5 months. We studied their behavior and the presence of α3, α4, α7, β2 and β4 nAChR subunits, Aβ_40_ and Aβ_42_ and activated astrocytes in the brain by sandwich ELISA and confocal microscopy. It was found that either LPS injections or immunizations with α7(1-208) resulted in region-specific decrease of α7 and α4β2 and increase of α3β4 nAChRs, accumulation of Aβ_42_ and activated astrocytes in the brain of mice and worsening of their episodic memory. Intravenously transferred α7 nAChR-specific-antibodies penetrated the brain parenchyma of mice pre-injected with LPS. Our data demonstrate that (1) neuroinflammation is sufficient to provoke the decrease of α7 and α4β2 nAChRs, Aβ_42_ accumulation and memory impairment in mice and (2) α7(1-208) nAChR-specific antibodies can cause inflammation within the brain resulting in the symptoms typical for Alzheimer disease.

## Introduction

Nicotinic acetylcholine receptors (nAChRs) are ligand-gated ion channels mediating fast synaptic transmission in muscle and autonomic ganglia [1]. In the brain, the nAChRs pre-synaptically control the release of several neurotransmitters, including dopamine, and influence cognition and memory, as well as establishment of nicotine dependence in smokers. In addition, nAChRs composed of α7 subunits are involved in regulating pro-inflammatory cytokines release in macrophages, brain astrocytes and microglia [2–5]. The decrease of the nAChR density in the brain neurons is observed upon Alzheimer disease (AD) [6], which is characterized by accumulation of oligomeric β-amyloids (Aβ) in the brain, memory impairments and loss of cognitive functions [7]. The AD is also accompanied by neuroinflammation, which often precedes the development of cognitive symptoms [8]. In spite of numerous investigations performed during the last decade the reason for the cholinergic deficit upon AD and its relation to neuroinflammation are still poorly understood.

Previously we found that antibodies raised against the extracellular epitopes of α7 nAChR subunit stimulated pro-inflammatory IL-6 production in cultured U373 glioblastoma cells [9] and were able to decrease the α7 nAChR density in certain brain regions to impair episodic memory of mice [10]. Such antibodies were found in the blood of both healthy humans and AD patients and seemed to be elevated in patients with the early-onset AD [11].

In the present study, we asked a question if systemic inflammation induced by regular injections of bacterial endotoxin (lipopolysaccharide, LPS) can provoke the AD-like symptoms in mice and if LPS effects can be mimicked by α7 nAChR-specific antibodies. The results demonstrate that either LPS injections or immunizations with α7(1–208) stimulated astrocyte activation, re-distribution of nAChR subtypes, accumulation of Aβ_42_ in the mouse brain and episodic memory impairment.

## Materials and Methods

### Ethics Statement

We used female C57BL/6J mice starting from 3 months of age. The mice were kept in the animal facility of Palladin Institute of Biochemistry, Kyiv. They were housed in a quiet, temperature-controlled room (22–23°C) and were provided with water and dry food pellets *ad libitum*. Before removing the brain mice were sacrificed by cervical dislocation. All procedures of this study including immunizations, blood collection and behavioural studies conformed to the guidelines and were approved by the Animal Care and Use Committee (IACUC) of Palladin Institute of Biochemistry, Kyiv Protocol 1/7-421.

### Reagents and antibodies

All reagents were of chemical grade and were purchased from Sigma-Aldrich unless specially indicated. Recombinant extracellular domain (1–208) of human α7 nAChR was produced as described [12]. Antibodies against α7(179–190), α3(181–192), α4(181–192), β2(190–200) and β4(190–200) nAChR fragments were obtained and characterized by us previously [13–14]. The following antibodies against Aβ have been used: mAb 4G8 (anti-Aβ_17–24_), mAb 11 A50 B10 (anti-Aβ_40_), mAb 12F4 (anti-Aβ_42_), all from Covance, USA; rabbit polyclonal antibody against glial fibrillary acidic protein (GFAP) was from Dako (Agilent Technologies); goat anti-rabbit IgG Alexa 488-labelled was from Invitrogen.

### Experimental model

A group of mice (10 animals) was immunized intraperitoneally with α7(1–208) (50 μg per mouse) and boosted every month during 5 months. The first two immunizations were performed with complete Freund’s adjuvant (CFA), the third one with incomplete Freund’s adjuvant, subsequent ones were done in PBS. Another group of mice (10 animals) was “immunized” with the adjuvant emulsified with PBS, similarly to group one, and then injected intraperitoneally with LPS (30 μg per mouse) instead of the antigen. The third group (5 animals) obtained adjuvant “immunizations” only. Control group of mice (10 animals) was intact.

After the end of immunization/treatment cycle, mice were examined in behavioral tests, and the blood from their tail vein was taken to measure the presence of α7(1–208)-specific antibodies. Then mice were sacrificed, their brains were removed and each brain was cut lengthwise into two halves: one half was homogenized with the glass homogenizer, while another one was fixed in 4% paraformaldehyde for 48 h, washed in PBS, dehydrated with increasing concentrations of alcohol and embedded in Paraplast X-TRA (McCormick Scientific LLC). The paraplast-embedded specimens were cut into serial frontal 5 μM sections with rotational microtome (HM 325, MICROM International GmbH). The sections were placed onto adhesive microscopic slides to be further examined by immunohistochemistry.

The primary brain homogenate was used to prepare the detergent lysate, as described previously [10]. Protein content was measured with the BCA kit (Thermo Scientific, France).

To study the antibody penetration into the brain 4 mice were injected intraperitoneally with LPS (30 μg per mouse) and the next day were injected intravenously with rabbit biotinylated either α7(1–208)-specific antibody or non-specific IgG (200 μg per mouse). Mice were sacrificed in 15 min and in 3h after the antibody injection; their brains were removed, fixed in 4% paraformaldehyde and cut by vibratome into coronal 40 μm sections. The floated sections were treated with Extravidin-Cy3 and placed onto microscopic slides to be examined by fluorescent confocal microscopy. Other 4 mice were injected with the antibody either with or without pre-treatment with LPS. They were also sacrificed in 15 min and in 3h after the antibody injection; their brains were homogenized in 0.4 ml of 0.02% NaN_3_-containing PBS and were centrifuged at 10,000 rpm for 10 min. The supernatants were collected, while the pellets were washed by another round of centrifugation and treated with 1% Tween-20-containing buffer as described previously [10]. The resulting lysate was cleared by centrifugation (13,000 rpm, 15 min). Both the primary supernatant and brain tissue lysate were used to determine the presence of biotinylated antibody by ELISA.

### ELISA assays

To determine the presence of α7(1–208)-specific antibodies in the mouse blood sera the immunoplates (Nunc Maxisorp) were coated with recombinant α7(1–208), 10 μg/ml in PBS, 2 h at 37°C, blocked with 1% BSA for 1 h at 37°C and washed by tap water before application of the sera in 0.05% Tween 20-containing PBS overnight at 4°C. The bound antibodies were detected with peroxidase conjugate of anti-mouse IgG and *o*-phenylendiamine-containing substrate solution; the optical density was read at 490 nm.

To determine the level of nAChR subunits within the brain lysates, the immunoplates were coated with rabbit α7(1–208)-specific antibody (20 μg/ml), blocked with 1% BSA, and the brain preparations were applied into the wells (1 μg of protein per 0.05 ml per well) for 2h at 37°C. The plates were washed with water and the second biotinylated α3(181–192)-, α4(181–192), α7(179–190)-, β2(190–200)- or β4(190–200)-specific antibody was applied for additional 2 h being revealed with Streptavidin-peroxidase conjugate and *o*-phenylendiamine-containing substrate solution.

To determine the level of Aβ within the brain lysates, the plates were coated with Aβ_17–24_-specific antibody (1:200), blocked with 1% BSA, and the brain preparations were applied as described above. The bound Aβ was revealed with biotinylated Aβ_40_- or Aβ_42_-specific antibody, Streptavidin-peroxidase conjugate and *o*-phenylendiamine-containing substrate solution. To determine the level of Aβ bound to α7 nAChR, the plates were coated with α7(1–208)-specific antibody, and the α7-Aβ complex from the brain preparation was revealed with biotinylated Aβ_40_- or Aβ_42_-specific antibody, Streptavidin-peroxidase conjugate and *o*-phenylendiamine-containing substrate solution.

To determine the presence of injected biotinylated α7(1–208)-specific antibody within the brain preparations of mice, the plates were coated with recombinant α7(1–208) and blocked with BSA as described above. The samples (either the primary supernatants or brain detergent lysates) were applied to the plates in several dilutions. The plates were incubated at 37°C for 2h and the bound antibody was revealed with Streptavidin-peroxidase conjugate and *o*-phenylendiamine-containing substrate solution.

### Immunohistochemistry and confocal microscopy

Before immunohistochemical staining, the brain sections were de-paraffinated by standard procedure; the non-specific binding was blocked with 1% BSA in PBS (30 min, RT). For staining the nAChR subunits, the slides were incubated with biotinylated α7(179–190)-, β2(190–200)- or β4(190–200)-specific antibodies overnight at RT, washed with PBS and incubated with Extravidin-Cy3 (1:200) and DAPI (1%) in 1% BSA-containing PBS for 1h at RT.

For staining the Aβ and α7 nAChR, the slides were incubated with both rabbit α7(179–190)-specific antibody and biotinylated mouse Aβ_40_- or Aβ_42_-specific antibodies revealed with Alexa-488-labeled goat anti-rabbit IgG and Extravidin-Cy3, respectively. Nonspecific binding was blocked with goat anti-mouse IgG.

For staining the astrocytes, the slides were incubated with rabbit anti-GFAP antibody followed by goat anti-rabbit-Alexa 488. The cell nuclei were stained with DAPI.

As negative controls, the incubations without primary antibody were performed in both control and experimental sections.

All slides with paraplast sections were embedded in MOWIOL-DABCO, while slides with floated brain sections were embedded in Vectashield with DAPI and examined under Zeiss LSM 510 Meta confocal laser scanning microscope. The brain regions were identified according to Paxinos and Franklin [15].

### Measuring IL-6

The primary supernatants of mouse brain homogenates were tested for the presence of IL-6 using the Murine IL-6 ELI-Pair kit from Diaclone (Gen-Probe, France), according to manufacturer’s instructions.

### Behavioral studies

Mice of all groups were tested in the “Open Field”, “Novel Object Recognition” (NOR) and “Elevated plus-maze” behavioral tests [16–18]. The results of NOR test are presented as Discrimination Index (DI) calculated as the difference in the number of “novel” and “famous” object explorations divided by the total number of explorations.

### Statistical analysis

ELISA experiments have been performed in triplicates and mean values for individual mice were used for statistical analysis assessed using the non-parametric Mann-Whitney test. Behavioral tests were also performed in triplicate for each mouse and mean values for individual mice were taken for statistical analysis. Correlation between the antibody level in the blood and the nAChR subunit level in the brain was calculated as Pearson coefficients for the OD_490_ values obtained in ELISA and Sandwich ELISA tests, correspondingly, in individual mice within experimental groups.

## Results

**Fig 1:**
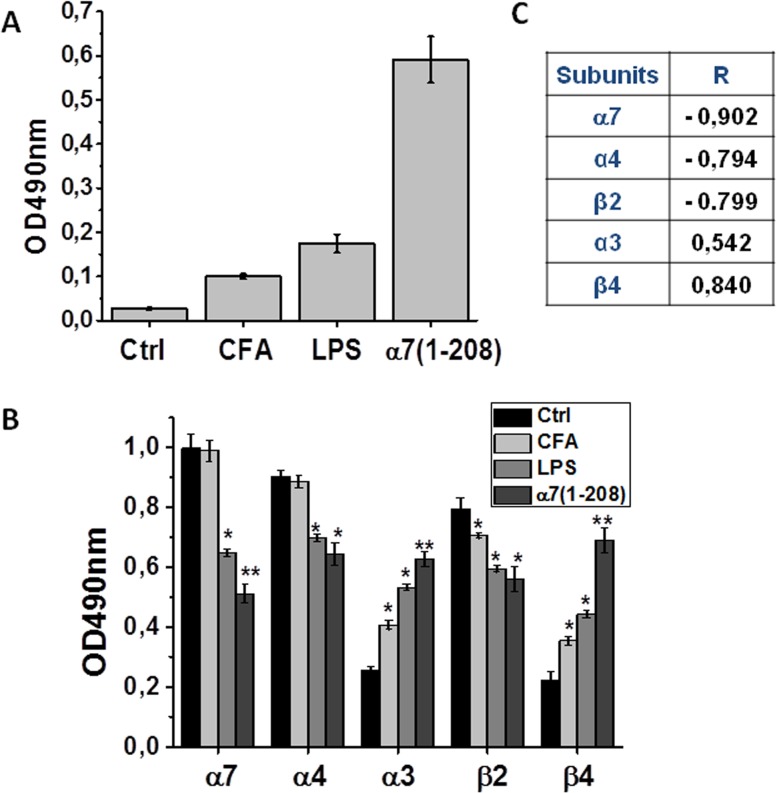
The level of nAChR-specific antibodies in the blood and of nAChR subtypes in the brain of experimental mice studied by ELISA (A) or Sandwich ELISA (B). **A**—7(1–208)-specific antibodies in the blood sera (1:50) of mice immunized with 7(1–208) (n = 8) or injected with LPS (n = 5) for 5 months compared to non-treated (Ctrl, n = 9) and adjuvant-“immunized” animals (CFA, n = 5). **B**—3, 4, 7, β2 and β4 nAChR subunits in the brain detergent lysates of the same groups of mice (4 mice from each group). **C**—Pearson coefficients (R) of correlation between the levels of nAChR subunits in the brain and those of 7(1–208)-specific antibodies in the blood. The columns correspond to M±SE, *—p<0.05; **—p<0.005; ***—p<0.0005 compared to Ctrl.

The blood sera of mice immunized with α7(1–208) contained significant amounts of antibodies against this nAChR fragment. Mice injected with LPS or “immunized” with the adjuvant only produced very low levels of α7(1–208)-specific antibodies, possibly, due to polyclonal B lymphocyte activation (Fig 1A).

### The level of different nAChR subtypes in the brains of α7(1–208)-immunized or LPS-treated mice

The brain preparations of both α7(1–208)-immunized and LPS-injected mice contained decreased amounts of α7, α4 and β2 and increased amounts of α3 and β4 nAChR subunits compared to control animals. Mice “immunized” with the adjuvant alone had slightly increased amounts of α3 and β4 subunits with no changes in α7 or α4 subunits (Fig 1B). Taking into account the established subunit combinations within the nAChR molecule, we could suggest that either immunizations with α7(1–208) or LPS injections resulted in the decrease of α4β2 and α7-containing nAChRs and the increase of α3β4 nAChRs, while stimulation with the adjuvant affected mainly α3β4 nAChRs. The levels of α4, α7 and β2 nAChR subunits negatively, while those of α3 and β4 subunits positively correlated with the levels of α7(1–208)-specific antibodies in the blood of individual α7(1–208)-immunized mice (Fig 1C). Therefore, redistribution of the nAChR subtypes upon immunization was obviously caused by the antibodies, but was antibody-independent in LPS-injected mice.

**Fig 2:**
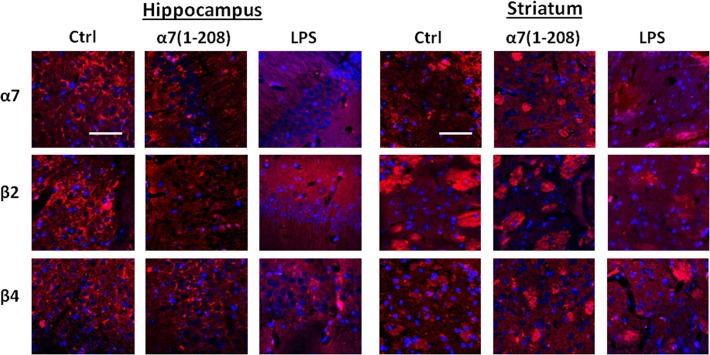
The level of α7, β2 or β4 nAChR subunits in the brain sections of experimental mice studied by immunohistochemistry. Confocal microscopy images of the hippocampus CA1 and striatum of non-treated (Ctrl), α7(1–208)-immunized or LPS-injected mice stained with biotinylated α7-, β2- or β4-specific antibodies and developed with Extravidin-Cy3 (*red*). Cell nuclei are stained with DAPI (*blue*). Bar corresponds to 50μm, actual for each fragment of the panel.

To determine, which brain areas were mostly affected with either the antibody or LPS, we performed immunohistochemical study of the brain sections of mice demonstrating the strongest changes in Sandwich ELISA. As shown in Fig 2, significantly weaker α7- and β2-specific staining with no visible changes in β4-specific staining were observed in hippocampal CA1 region of α7-immunized and LPS-injected mice compared to controls. In striatum, immunization with α7(1–208) did not affect significantly α7-, obviously decreased β2- and increased β4-specific staining, while LPS-induced inflammation seemed to decrease both α7- and β2- and to increase β4-specific staining. In contrast to the hippocampus, where the antibodies stained the cell bodies, the nerve bundles mainly were stained in striatum. No significant changes in α7, β2 or β4 nAChR subunits were found in the motor/somatosensory cortex (data not shown).

The α7 nAChRs have been classically described as homopentamers [1, 6]. However, more and more data appear to show that α7 subunits can be combined with β2 subunits in the brain [19] and, in particular, in the hippocampus [20]. According to the data presented in Fig 2, we could expect the decrease of both α7 and α7β2 nAChRs in the hippocampus and striatum of both immunized and LPS-treated mice.

The decrease of α7- and α4-containing nAChRs was accompanied by the increase of α3β4 nAChRs. This finding is in accord with the previously published data [21] showing that inhibition of the brain α7 nAChR activity with kynurenic acid resulted in an increase in non-α7 nicotinic receptor expression. Previously we observed the increase of α4β4-containing nAChRs in the brains of mice immunized with α7(1–208) during 2 months and the increase of β4-containing nAChRs in α7-/- mice [10]. This data indicate that β4-containing receptors are the established alternate α7-substituting nAChR subtype(s); the antibody-mediated α7 nAChR decrease is at first compensated with α4β4 nAChRs, while prolonged antibody presence, as well as chronic inflammation induced by regular LPS injections, also decrease α4-containing receptors compensated with α3β4 ones.

Knock-out studies suggest that nAChRs containing β2 or β4 subunits play different roles in mouse brain [22–23]. The results of studies presented below also demonstrate that α3β4 receptors, which potentially substitute the α7 and α4β2 receptors, are functionally not equal to them.

### **The** Aβ **isoforms in the brains of α7(1–208)-immunized or LPS-treated mice**

**Fig 3:**
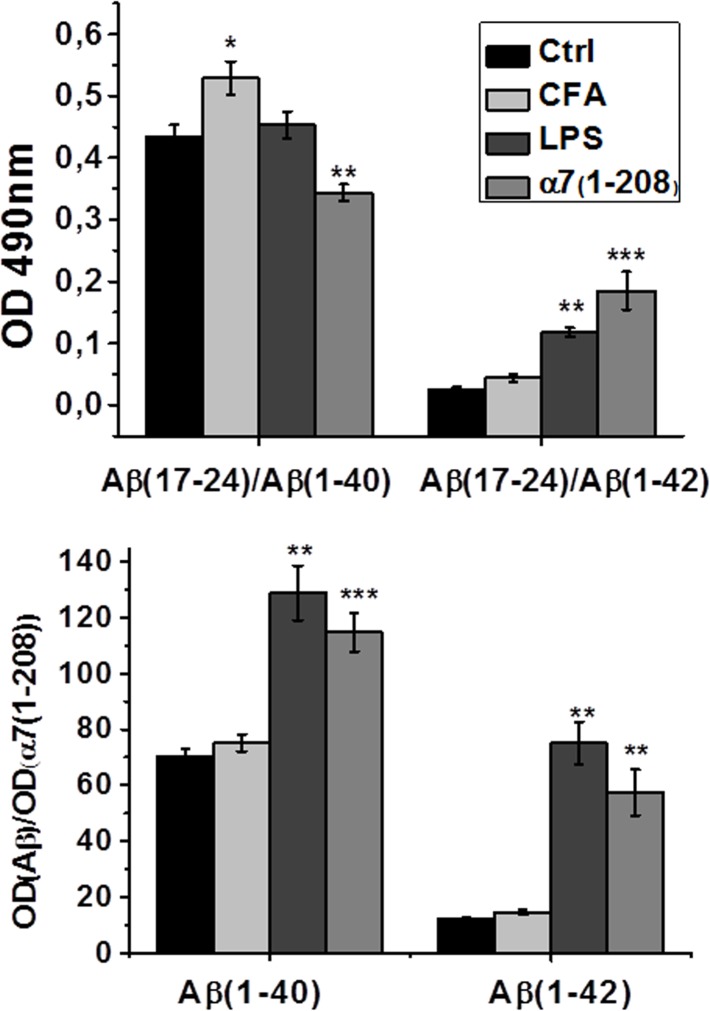
The levels of different Aβ isoforms in the brain detergent lysates of experimental mice studied by Sandwich ELISA. **A**—total Aβ_40_ and Aβ_42_, **B**—Aβ_40_ and Aβ_42_ bound to α7 nAChR in mice immunized with 7(1–208) (n = 8) or injected with LPS (n = 5) compared to non-treated animals (Ctrl, n = 9) and to those “immunized” with complete Freund’s adjuvant (CFA, n = 5). The columns correspond to M±SE (n = 5); *—p<0.05; **—p<0.005; ***—p<0.0005 compared to Ctrl.

The α7 nAChR subtype is known to be involved in the Aβ processing and metabolism [24]. The processing of amyloid precursor protein results in two alternative isoforms: 1–40 (Aβ_40_) or 1–42 (Aβ_42_), from which the Aβ_42_ is considered more toxic than the Aβ_40_ [25]. The wild-type mice do not form insoluble amyloid plaques [26]; however, the soluble Aβ processed peptides can be determined. We measured them by Sandwich ELISA in the brain preparations of experimental mice. As shown in Fig 3A, the level of Aβ_40_ obviously decreased after immunization with α7(1–208), while the level of Aβ_42_ increased upon both inflammation and immunization.

In the next version of Sandwich ELISA, were the antigen was captured with α7-specific antibody and revealed with Aβ-specific antibody, we measured the quantity of Aβ_40_ and Aβ_42_ coupled to α7 nAChR. The resulting signal was normalized to that of α7. As shown in Fig 3B, the part of both Aβ_40_ and Aβ_42_ bound to α7 nAChR increased after either immunization or LPS stimulation. Control “immunization” with CFA slightly increased both Aβ_40_ and Aβ_42_ and did not affect their interaction with α7.

**Fig 4:**
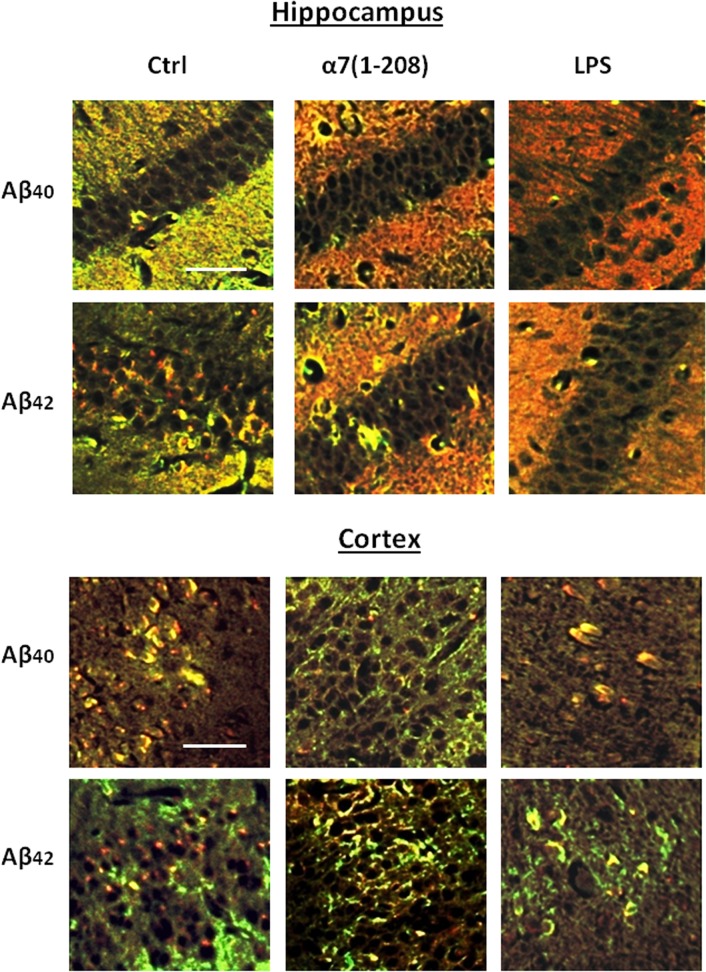
The level of Aβ_40_ and Aβ_42_ in the brain sections of experimental mice studied by immunohistochemistry. Confocal microscopy images of the hippocampus CA1 or motor/somatosensory cortex of non-treated (Ctrl), α7(1–208)-immunized or LPS-injected mice stained with biotinylated Aβ_40_- or Aβ_42_-specific antibodies and developed with Extravidin-Cy3 (*red*) and with α7-specific antibody developed with anti-rabbit Alexa 488 (*green*). Bar corresponds to 50μm, actual for each fragment of the panel.

Aβ accumulations can also be identified immunohistochemically [27]. We observed the Aβ_42_ as local granules in pyramidal cell layer of the hippocampus and in the motor/somatosensory cortex of non-treated animals. In α7-immunized and LPS-injected mice the staining spread out and surrounded the cells in a diffuse way merging with that for α7 (Fig 4). The Aβ_40_ isoform was found out of pyramidal layer in the hippocampus, and its quantity increased after either α7 immunizations or LPS injections. In contrast, in the cortex, Aβ_40_-specific staining was initially merged with α7-specific one and obviously decreased after immunization, correlating with the data of Sandwich-ELISA (Fig 3A). No definite changes of Aβ were found in the striatum (data not shown).

### The inflammatory markers in the brains of α7(1–208)-immunized or LPS-treated mice

**Fig 5:**
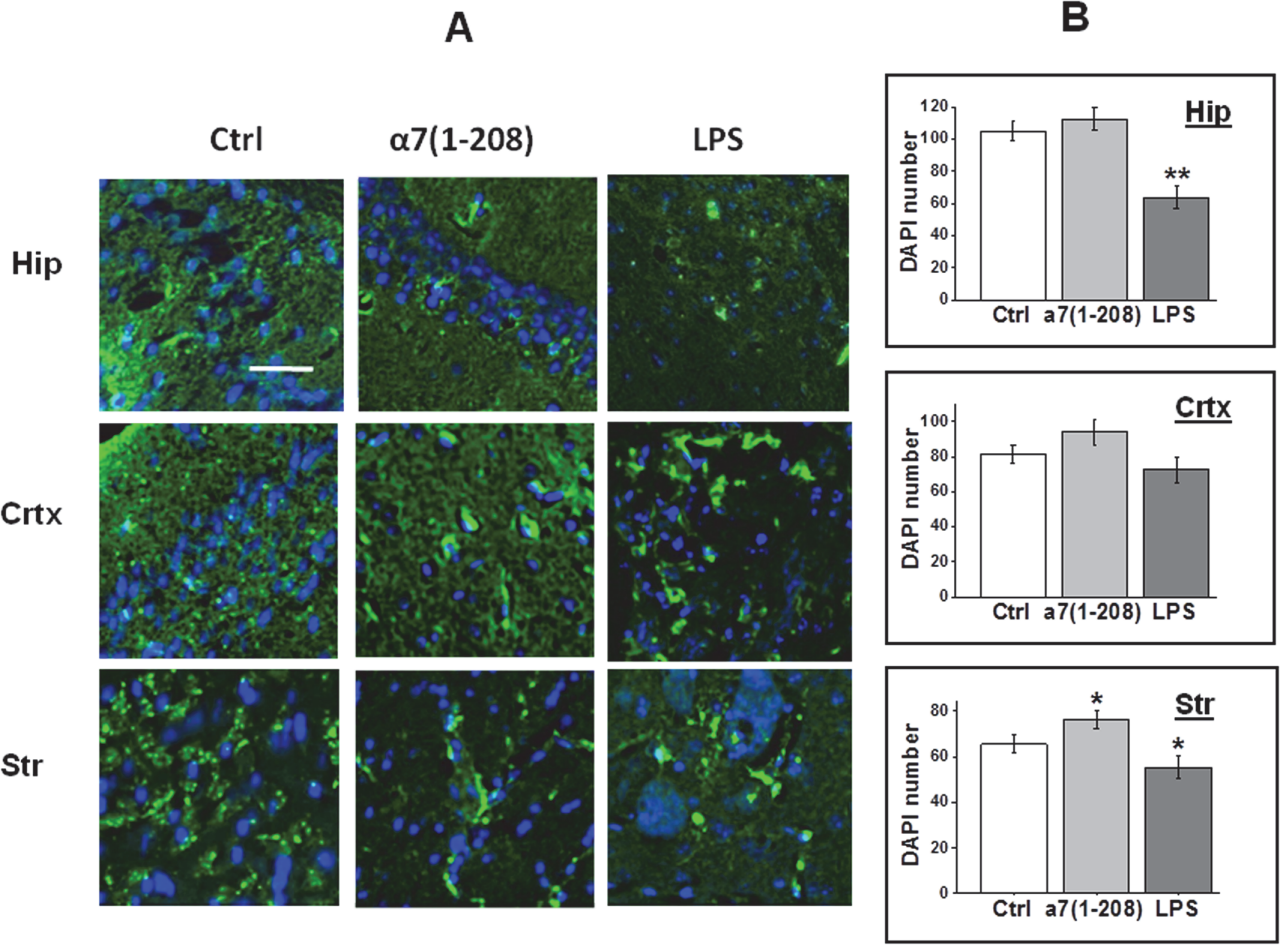
The GFAP-positive astrocytes (A) and the number of nucleated cells (B) in the brain sections of experimental mice studied by immunohistochemistry. **A**—Confocal microscopy images of the hippocampus CA1 (Hip), motor/somatosensory cortex (Crtx) or striatum (Str) of non-treated (Ctrl), α7(1–208)-immunized or LPS-injected mice stained with rabbit GFAP-specific antibody and developed with anti-rabbit Alexa 488 (*green*). Cell nuclei are stained with DAPI (*blue*). Bar corresponds to 50μm, actual for each fragment of the panel. **B**—The number of nucleated cells (DAPI-positive) in corresponding brain regions studied in all available sections (12 to 16 for each treatment for each region); *—p<0.05; **—p< 0.005.

We looked for accumulation of glial fibrillary acidic protein (GFAP), a recognized marker of astrocyte activation [28] in the brain sections of experimental mice. As shown in Fig 5A, the GFAP-specific antibody stained small weakly activated astrocytes in the control cortex, hippocampus and striatum. Either immunizations with α7(1–208) or LPS injections resulted in intensive GFAP-specific staining and appearance of large GFAP-positive astrocytes in the motor/somatosensory cortex and striatum. The weakest astrocyte reaction was found in the hippocampus where α7 nAChRs were mostly decreased.

We also measured the interleukin-6 (IL-6) levels in the primary supernatants of homogenized brains. The signal appeared to be very low; however, it was significantly elevated (p<0.05) in both LPS-injected (OD_490_ = 0.04±0.005) and, especially, in α7(1–208)-immunized mice (OD_490_ = 0.055±0.008) compared to control animals (OD_490_ = 0.021±0.003).

The data presented in Figs 2 and 5A seemed to demonstrate that the brain preparations of antibody- and, especially, LPS-treated mice contained less nucleated cells compared to control sections. We performed statistical calculations of DAPI-positive cells in all available images. The result presented in Fig 5B indicates that LPS treatment indeed decreased the number of cells in the hippocampus and striatum, whereas immunization even slightly increased it.

### Study of the antibody penetration into the brain parenchyma

**Fig 6:**
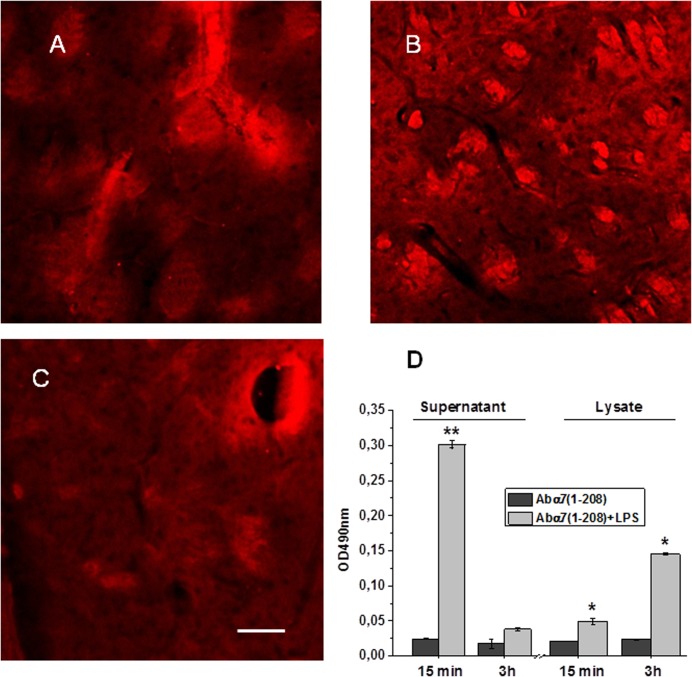
Visualization of biotinylated antibody within the brain at different periods after injection. Confocal microscopy images of the striatum of mice injected with α7(1–208)-specific antibody (A-B) or non-specific IgG (C) in 15 min (A) or 3h (B-C) after injection. Antibodies were developed with Extravidin-Cy3 (*red*). Bar corresponds to 100μm, actual for each fragment of the panel. D—Antibody-specific ELISA signal of the primary brain supernatants and detergent lysates of mice either pre-treated or not with LPS; the brains were removed in 15 min or 3 h after the antibody injection. Each column corresponds to mean±SE of three repeats in ELISA; *—p<0.05; **—p<0.005 compared to the data of LPS non-treated mice.

The observed α7(1–208)-specific antibody effects naturally put a question if the antibody penetrated the brain and affected the brain cells directly or it stimulated the inflammatory reaction in the periphery to mimic LPS effect. To test this we injected mice with biotinylated α7(1–208)-specific antibody, removed their brains at various periods after injection and examined them by confocal microscopy after being stained with fluorescently labeled Extravidin. To compromise the blood-brain barrier, mice were pre-treated with LPS a day before the antibody injection. As shown in Fig 6A, the conspicuous fluorescent signal was observed within and close to the blood vessels of striatum already in 15 min after the antibody injection. After 3 hours, the staining spread out into parenchyma and was concentrated around the nerve bundles and cells (Fig 6B). The non-specific IgG penetrated the brain parenchyma much weaker and remained distributed around the blood vessel in 3 h after injection (Fig 6C). The obvious but less intensive staining was also observed in the hippocampus and cortex (data not shown). This data clearly indicated that α7(1–208)-specific antibody penetrated the brain and bound specifically to α7-positive cells and that striatum was one of the primary targets of these antibodies.

We also tested if the antibody could penetrate the brain given the blood-brain barrier was not damaged. For this purpose, the brains of mice injected with biotinylated α7(1–208)-specific antibody with or without LPS pre-treatment were fractionated into the primary supernatant and brain tissue detergent lysate (see Methods). It was expected that the supernatant contained the antibody, which penetrated the brain but did not bind to any brain structures; it could also include the antibody within small blood vessels of the brain. In contrast, the detergent lysate was expected to contain the antibody bound to the brain tissue. As shown in Fig 6D, significant antibody signal was found in the supernatant obtained in 15 min after the antibody injection in LPS-pre-treated mouse; it decreased dramatically after 3h. In contrast, the signal from the detergent lysate increased about 3-fold from 15 min to 3 h. No signal was found in any preparation of mice not treated with LPS. This data obviously indicated that α7(1–208)-specific antibody could not penetrate the brain if the blood-brain barrier had not been compromised by inflammation. In LPS-treated mice, the antibody penetrated the brain parenchyma during 15 min after injection and remained bound to the brain structures for at least 3 h.

### Behavior changes in α7(1–208)-immunized or LPS-treated mice

**Fig 7:**
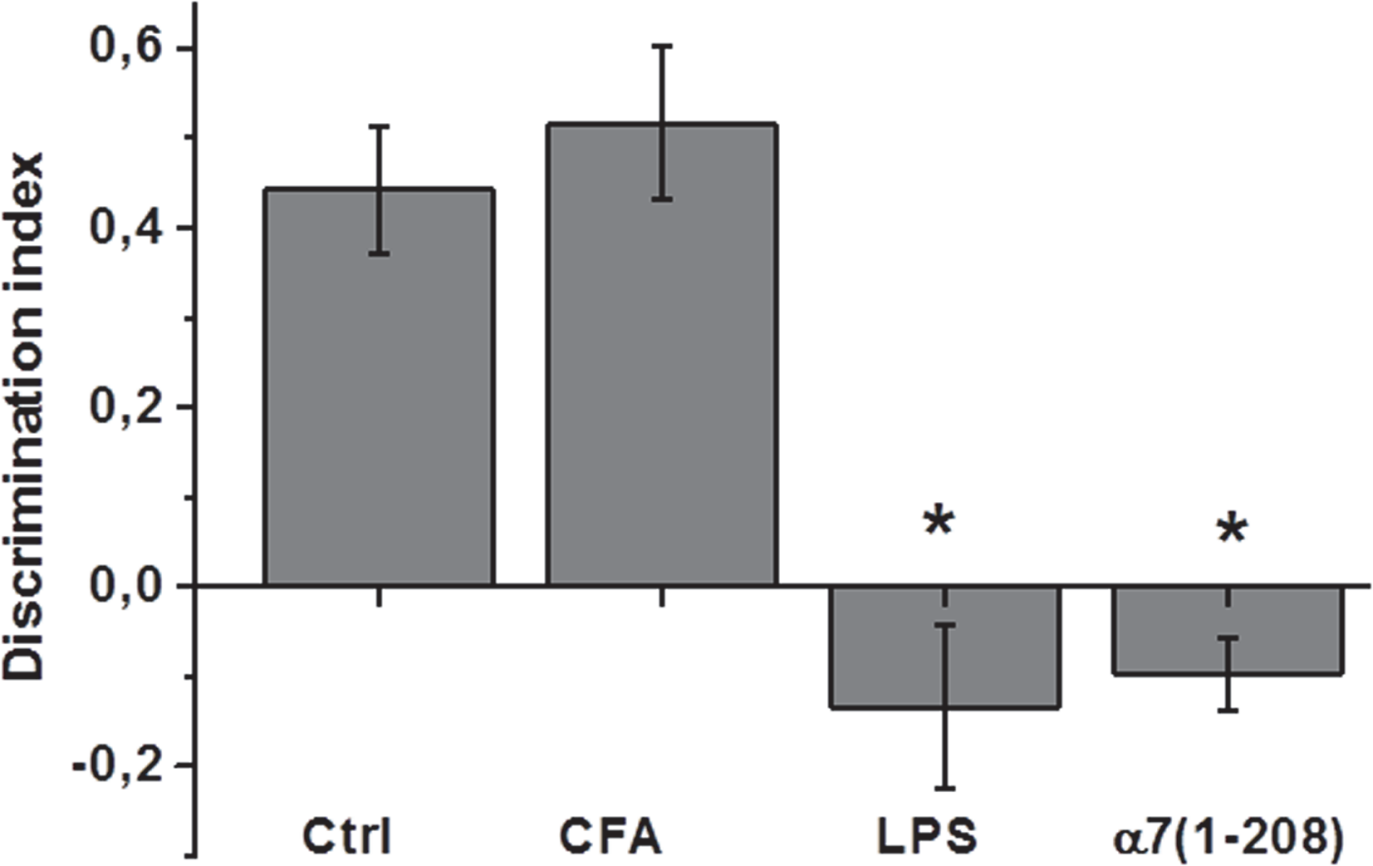
Episodic memory of experimental mice studied in the “Novel Object Recognition” test. Discrimination indexes calculated for mice immunized with 7(1–208) (n = 8) or injected with LPS (n = 5) compared to non-treated animals (n = 9) or those “immunized” with complete Freund’s adjuvant (CFA, n = 5). ***—p<0.0005 compared to non-treated mice (Ctrl).

The behavioral studies demonstrated that both α7(1–208)-immunized and LPS-injected mice significantly differed from non-treated and CFA-immunized animals in the NOR test related to episodic memory (Fig 7). No significant differences were found in locomotor or anxiety-related activities (the “Open-field” test), as well as in “Elevated plus-maze” behavioral test (data not shown).

## Discussion

The results presented here demonstrate a striking similarity of effects exerted by LPS injections or immunizations with α7(1–208) in mice. Both treatments resulted in redistribution of nAChR subtypes, accumulation of Aβ_42_ and inflammatory reaction within the brain, as well as significant memory impairment. This similarity could not be explained by the adjuvant stimulation during immunizations, because control injections of adjuvant did not produce such effects. Moreover, in our previous experiments, mice immunized with BSA using similar immunization schedule did not demonstrate such profound changes in the nAChR content within the brain and in behavior as α7(1–208)-immunized mice [10]. The data presented here help to understand the mechanism of the nAChR-specific antibody action within the brain, as well as the role of inflammation in the development of AD-like symptoms.

According to our previous results, the 2-month immunizations with α7(1–208) decreased the density of α7 nAChRs mainly in striatum and discrimination index in the NOR test was about 0.2 [10]. As shown here, after 5 months of immunizations, the decline of α7- and β2-containing nAChRs was found mainly in the hippocampus resulting in dramatic worsening of episodic memory: discrimination index in the NOR test was less than -0.1. Therefore, prolonged presence of α7(1–208)-specific antibodies in the brain resulted in spreading their effects and significant aggravation of memory deficit.

The antibody can penetrate the brain only if the blood-brain barrier has been compromised. LPS injected intraperitoneally stimulates peritoneal macrophages to produce pro-inflammatory cytokines like IL-1β, IL-6 and TNFα [29–30], which are transported to the brain with the blood flow and favor loosening the tight junctions between the brain vascular endothelial cells [31]. Previously we found that α7(1–208)-specific antibodies injected into mice intravenously affected the nAChR levels in the brain only if mice were additionally injected intraperitoneally with LPS. In case of immunization, the effect was much stronger if the antigen was introduced intraperitoneally with complete Freund’s adjuvant, which includes LPS-containing micobacteria [10]. In the present paper, we demonstrate that the antibodies do penetrate into the brain parenchyma and can directly bind α7-bearing structures only if mice were pre-treated with LPS. The intensive staining found in striatum suggests that this brain region is one of the first targets of the antibodies crossing the blood-brain barrier. The changes in the nAChR subunits within the brain correlated with the antibody levels in the blood of individual mice indicating the obvious involvement of the antibody in this process.

Hippocampus is known to control episodic memory [32], while striatum is responsible for maintaining procedural memory [33]. The two memory systems work in parallel [34] and both are dependent on α7 nAChRs [35–40]. The hippocampus projects to the ventral striatum underlying functional interactions between these two regions [40]. Taking into account that α7 nAChRs were found mainly on the striatal nerve bundles (Fig 2), their decrease after 2-months immunizations could prevent the hippocampus-striatum interaction thus affecting episodic memory.

The hippocampus-dependent memory is also influenced by Aβ peptides [41], which were shown to bind with both α7 and α7β2 nAChRs [42]. Here we demonstrate that the decrease of α7(β2) nAChRs upon either immunizations or LPS injections was accompanied by accumulation of Aβ_42_ and Aβ_40_ in the hippocampus, spreading Aβ_42_ out of pyramidal cell layer and its combination with the remaining α7. No Aβ accumulation was observed in striatum where α7 nAChRs were not decreased. Accumulation of Aβ_42_ could contribute to the dramatic decline of episodic memory observed in α7(1–208)-immunized or LPS-injected mice. It is not clear whether increased Aβ-α7 interaction was pathogenic or defensive towards Aβ_42_ toxicity. Our data also demonstrate that depletion of α7 nAChRs within the brain upon inflammation or under the antibody effect was sufficient to trigger Aβ_42_ accumulation and memory impairment. Memory deficit has been reported in mice chronically treated with LPS [43] that agrees with our observation.

α7 nAChR is now commonly recognized to mediate anti-inflammatory effect of acetylcholine in macrophages [2–3], microglial cells [4] and astrocytes [5]. We observed the decreased α7 nAChR level, increased IL-6 level and GFAP-positive astrocyte activation in the brains of both α7(1–208)-immunized and LPS-injected mice. The decreased expression of α7 nAChRs in cultured cells under chronic presence of inflammatory cytokines has been reported [44], however, its mechanism is not known. The antibody could facilitate the α7 nAChR catabolism by binding and internalizing the plasma membrane receptors [9], and the accompanying decrease of α4-containing nAChRs could be due to certain cross-reactivity of α7(1–208)-specific antibody because of substantial homology of extracellular domains of α7 and α4 subunits [1].

Previously we found that α7-specific antibody stimulated IL-6 production in cultured U373 glioblastoma cells [10]. In addition, Aβ_42_ has been shown to induce cyclooxygenase-2 activation in astrocytes resulting in pro-inflammatory cytokine production [45]. Therefore, the α7(1–208)-specific antibody could induce inflammation in the brain either directly or by decreasing the α7 nAChR expression that prevented anti-inflammatory effect of acetylcholine and stimulated Aβ_42_ accumulation. Probably, severe inflammation induced by Aβ_42_ resulted in cell depletion in the hippocampus and striatum of LPS-injected mice (Fig 5B). However, this was not the case in α7(1–208)-immunized animals, possibly, due to described protective role of α7-specific antibody against Aβ cytotoxicity [46]. Therefore, the antibody seemed to play a dual role within the brain: it stimulated Aβ accumulation but supported cell viability that is in accord with the documented pro-survival role of α7 nAChRs [47].

## Conclusions

The data presented here allow concluding that (1) systemic inflammation is sufficient to provoke the decrease of α7 nAChRs, Aβ_42_ accumulation in the brain and memory impairment and (2) α7 nAChR-specific antibodies can induce inflammation within the brain. Taking into account that the antibodies penetrate the brain only if the blood-brain barrier has been loosened by inflammation, we raise a hypothesis shortly expressed as “Inflammation procreates inflammation”. According to it, peripheral inflammation facilitates penetration of α7-specific antibodies into the brain where they stimulate neuroinflammation, α7 nAChR decrease, Aβ accumulation and memory impairment.

Inflammation is a common feature of Alzheimer disease [48–50]. Moreover, both experimental and clinical data suggest that inflammation precedes cognitive impairments and may stimulate their development [8] that is in good agreement with our data. Therefore, inflammation, classically considered as a side symptom of AD, appears to be one of its primary reasons. Consequently, regular injections of LPS used in our studies can be suggested as a way of creating an AD model in the wild-type mice.

Previously we reported the presence of α7(1–208)-specific autoantibodies in the blood sera of healthy humans; their level increased with age reaching maximum at about 20 years old. It was elevated in children suffering from the acute form of obstructive bronchitis; therefore, we suggested that the antibodies were produced in response to destructed bronchial epithelial cells known to express α7 nAChRs [51]. This suggestion was confirmed when we observed the production of α7(1–208)-specific antibodies in mice injected with Lewis lung carcinoma cells originating from lung epithelium. In the same study, it was shown that patients with the early-onset Alzheimer disease possessed elevated levels of α7(1–208)-specific antibodies compared to those with the late-onset dementia [11]. The results presented here allow suggesting the high α7(1–208)-specific antibody titer combined with the frequent inflammatory diseases facilitating their penetration into the brain as one of the risk factors for the development of cognitive symptoms of Alzheimer disease like memory loss.
